# One in Four US Households Likely Exceed New Soil Lead Guidance Levels

**DOI:** 10.1029/2024GH001045

**Published:** 2024-06-18

**Authors:** Gabriel M. Filippelli, Matthew Dietrich, John Shukle, Leah Wood, Andrew Margenot, S. Perl Egendorf, Howard W. Mielke

**Affiliations:** ^1^ Department of Earth and Environmental Sciences and Environmental Resilience Institute Indiana University Indianapolis IN USA; ^2^ ZevRoss Spatial Analysis Ithaca NY USA; ^3^ Department of Crop Sciences University of Illinois Urbana‐Champaign Urbana IL USA; ^4^ Department of Environmental Studies and Science Pace University New York NY USA; ^5^ Affiliate, Tulane University School of Medicine New Orleans LA USA

**Keywords:** lead, soil, contamination, lead poisoning, USEPA

## Abstract

Lead exposure has blighted communities across the United States (and the globe), with much of the burden resting on lower income communities, and communities of color. On 17 January 2024, the US Environmental Protection Agency (USEPA) lowered the recommended screening level of lead in residential soils from 400 to 200 parts per million. Our analysis of tens of thousands of citizen‐science collected soil samples from cities and communities around the US indicates that nearly one quarter of households may contain soil lead that exceed the new screening level. Extrapolating across the nation, that equates to nearly 30 million households needing to mitigate potential soil lead hazards, at a potential total cost of 290 billion to $1.2 trillion. We do not think this type of mitigation is feasible at the massive scale required and we have instead focused on a more immediate, far cheaper strategy: capping current soils with clean soils and/or mulch. At a fraction of the cost and labor of disruptive conventional soil mitigation, it yields immediate and potentially life‐changing benefits for those living in these environments.

## Key Points

- The US Environmental Protection Agency recently reduced its soil lead screening recommendations
- We find that nearly one in four households may now contain a soil lead hazard based on the new recommendations
- This finding challenges the current expensive approach, sometimes termed "dig and dump," to mitigation at this scale

## 1 Introduction

**Figure 1:**
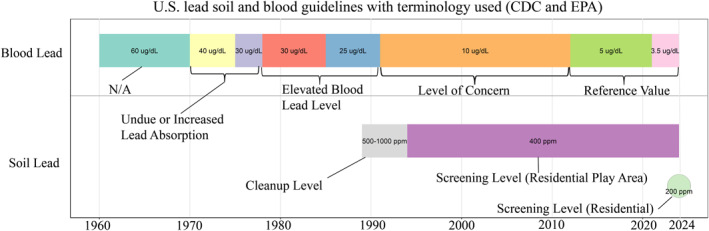
Blood and soil lead guidelines issued from the U.S. CDC and EPA over time, with labels representing the terminology used by the corresponding agency for each respective guideline.

Unsafe levels of lead exposure has occurred in many communities across the United States, with much of the burden resting on lower income communities, and communities of color. Lead exposure is prevalent due to past lead emissions and the substantial legacy lead loads that remain in soils and structures within communities The reason behind the disproportionate exposure are myriad, including Redlining and other societal shifts during the twentieth century, but the results are manifest in lower educational outcomes and lower economic potential for exposed communities. Despite immense current federal efforts to “get the lead out,” our national lead problem is nowhere near over (Figure 1).

With the Clean Air and Clean Water Acts, US policy began severely limiting the production and use of lead in infrastructure and consumer products, including water pipes, paint, and gasoline that have been historical sources of lead entry into the environment. The results have been stunningly successful and positive overall—with a decline in the percentage of children affected by lead (by modern standards) from nearly 100% of the population in the 1970s to about 1% today. However, these improvements in health outcomes are not shared equally, and many urban children are still exposed to lead at unsafe levels. Notably, this continuing toxic exposure does not primarily come from the pipes, paint, and gasoline targeted by the Clean Air and Water Acts. Degradation of aging lead‐based paint on pre‐1980s housing, and past deposition from leaded gasoline and industrial emissions, means that urban lead is now in the soils and dust upon which neighborhoods are built, children play, and food is grown. In other words, our modern lead problem is a legacy of historical contamination: the lead is still largely present the environment and posing continued risks to communities.

In recognition of this continued exposure from soil, and the dust generated from those contaminated soils (e.g., Laidlaw et al., 2012), on 17 January 2024, the US Environmental Protection Agency (USEPA) has, more than 30 years after setting the 400 parts per million (ppm or mg/kg) guidance, lowered the recommended level of lead in residential soils (USEPA, 2024a). The original 400 ppm screening level was based on the science of the time, linking contaminated soils to average blood lead levels of children living in these locations. While the Centers for Disease Control and Prevention has continuously ratcheted down the blood lead standard for children's health, from 10 μg per deciliter in the 1990s to the current 3.5 μg per deciliter health safety standard, the soil standard has remained unchanged, until now. The population‐based approach for blood lead standards is not without its challenges (e.g., Roy et al., 2023), particularly as we are approaching lower general blood lead levels in children, but it has reflected population‐based trends, unlike the soil recommendations. In recognition of this public health prevention mismatch, several states, including California, have adopted their own screening level for soils. For example, California's soil lead screening level is 80 mg/kg, set as a 90th‐percentile estimate of a 1 μg/dL increase in the blood lead of a child (DTSC, 2019). Why then has the USEPA soil lead recommendations lagged behind? Based on our research on a broad range of household soil samples, we propose that it is simply the immensity and ubiquity of the problem.

## 2 Results and Discussion

Our analyses of tens of thousands of citizen‐science collected soil samples from cities and communities around the US (MapMyEnvironment, 2024) and supplemented by similar sample sets from colleagues around the country reveal the scale of the soil lead problem. These samples don't come from Superfund clean‐up sites, but rather from the soil around real residential properties nationwide that people live in and call home.

The new USEPA soil screening level has been set at 200 ppm for residential properties. At residential properties with multiple sources of lead exposure, the USEPA will generally use 100 ppm as the recommended level. This is heading closer to the levels adopted by California (80 ppm) and those of many other countries. The change is welcomed by environmental health professionals around the country, as it reflects our knowledge of today's major lead exposure sources being largely soil‐based. But our analysis of the on‐the‐ground reality suggests why it has taken the USEPA awhile to lower this protective guideline—namely, once the threshold for lead in soils is lowered, the agency needs to consider providing guidance and resources to every household whose soils exceed the new threshold. The scale is astounding, and the nation's lead remediation efforts just became substantially more complicated.

Our nationwide analysis of our citizen science database of 15,595 residential soil samples reveals that in the US, just over 12% of residentially collected soils (including from yards, gardens, driplines, alleys, etc.) exceed the previous standard of 400 ppm (Table 1; Figure 1). This alone is a startling finding, but when the recommendation is decreased to the new 200 ppm screening level, nearly one quarter of households contain a lead hazard. Extrapolating across the nation, that equates to roughly 29 million households (out of 123.6 million total based on the 2020 census) needing to mitigate potential soil lead hazards. As that level drops to the screening level of 100 ppm for residences with multiple lead exposure sources, which just over 40% of household soil samples exceed, the number goes up to nearly 50 million households. These aggregated results are largely via XRF analysis in the laboratory and reflect total lead concentrations—given variations in lead bioaccessibility in various soils, the human‐absorbable fraction of lead from soil will be lower.

**Table 1:** Percent and Number of Household Soil Samples That Fall Within Various Soil Lead Concentration Bins

	*n*	Soil lead (mg/kg)							
		≥400		200 to <400		100 to <200		<100	
		%	*n*	%	*n*	%	*n*	%	*n*
United States	15,595	12.3	1,933	11.4	1,789	15.7	2,475	59.8	9,398
New Orleans, LA	5,434	14.8	805	10.4	566	13.2	718	61.6	3,345
South Bend, IN^a^	4,905	4.3	216	5.0	249	12.0	604	76.4	3,836
Indianapolis, IN	2,641	20.1	530	17.0	448	20.1	531	42.8	1,132
Chicago, IL	1,187	20.2	240	32.6	387	29.5	350	17.7	210
Burlington, VT	523	17.8	94	5.9	31	11.8	62	63.8	336
Springfield, MA	166	3.6	6	11.9	20	26.8	45	56.5	95
Lowell, MA	152	3.9	6	12.5	19	38.8	59	44.7	68
Hartford, CT	139	10.7	15	21.4	30	32.9	46	34.3	48
Winston‐Salem, NC^b^	53	15.1	8	20.8	11	26.4	14	37.7	20
Chattanooga, TN^b^	53	5.7	3	5.7	3	15.1	8	73.6	39
Louisville, KY^b^	53	5.7	3	22.6	12	20.8	11	50.9	27
Memphis, TN^b^	53	5.7	3	9.4	5	11.3	6	73.6	39
Lexington, KY^b^	53	3.8	2	7.5	4	17.0	9	71.7	38
Columbia, SC^b^	53	0.0	0	1.9	1	5.7	3	92.5	49
Raleigh, NC^b^	53	0.0	0	0.0	0	5.7	3	94.3	50
Gainesville, FL^b^	53	0.0	0	0.0	0	1.9	1	98.1	52

^a^ Samples taken in a gridded pattern throughout St. Joseph County, and thus represent a mix of urban, suburban, and rural sampling locations.

^b^ Samples specifically collected to represent “urban background” locations, and thus not near typical urban lead sources (USEPA, 2023).

**Figure 2:**
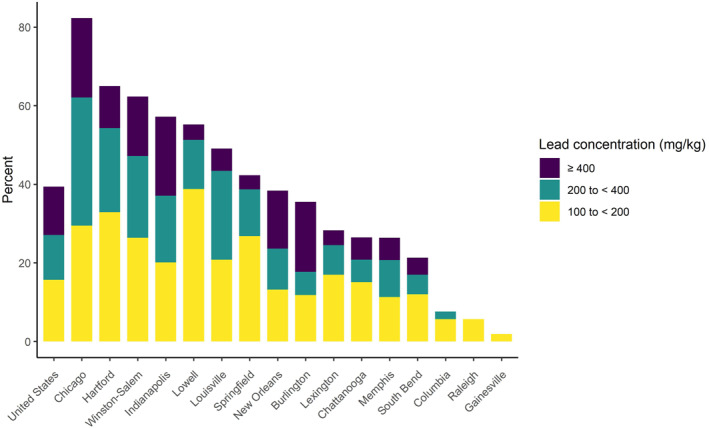
Percentage of samples within each city and the United States from Table 1 that exceeded 100 mg/kg lead, binned by concentration category.

The integrated data set upon which these analyses were conducted does include some differences in sampling protocols, sampling dates, and sampling dates for various municipalities. For example, the cities of Chicago, Indianapolis, and New Orleans utilized similar citizen‐science approaches for collecting large numbers of samples that represent community‐scale lead distribution, which we feel is highly representative of household exposure potential. One outcome of this approach is that these municipalities have a much higher percentage of household soils that have high lead values, including Chicago with 53% of household soils above the new 200 ppm recommendation (Table 1; Figure 2). Meanwhile, other municipalities have soil samples that are either collected by researchers to identify hotspots and backgrounds (i.e., South Bend) or were specifically focused on urban background locations (Table 1). Samples were collected at various times over the past 15 years, and lead concentrations might have changed due to land use practices, disturbance, and/or new additions of lead (e.g., from deteriorating lead‐based paints). It is thus difficult to fully assess city‐specific soil lead burdens, both because of the limitations of the citizen science data set upon which this analysis is based (i.e., uneven sampling throughout a city) and because there is no other systematic, comprehensive measurement of residential soil lead values across the US.

Our citizen science initiatives are intended to empower people with knowledge about lead exposure risks at their homes and in their neighborhoods, and to engage them in the development of simpler approaches and test kits for soil lead assessment (Dietrich et al., 2022). While these initiatives have provided us with far more data points than we could have collected ourselves, the voluntary nature of citizen science means that the distribution of data points is not homogenous. In metro areas with high levels of participation (New Orleans, LA; South Bend, IN; Indianapolis, IN; Chicago, IL), we can be more confident in our assessment of the overall soil lead burden than in metros where data is scarce. Likewise, our extrapolations of the numbers across the US are subject to the limitations of the data set and should therefore be considered preliminary. We anticipate that the new USEPA soil lead screening level of 200 ppm will raise awareness of the ongoing lead problem and increase participation in our citizen science initiatives. This, in turn, will grow our data set and improve projections. In the meantime, these preliminary results are nevertheless illustrative of the massive scale of the task ahead in light of the new USEPA soil standard.

These results indicate that soil lead in residential neighborhoods can be an issue, and we don't really know which particular households, besides older homes being a general risk factor (e.g., Dietrich, Wood, et al., 2023), have the greatest risk potential This is due both to the paucity of publicly available data for lead and to the very small spatial scale at which lead hotspots occur—even within a household itself (Dietrich, Rader, & Filippelli, 2023). Thus, the real cost of mitigating this soil lead problem is unknown. At a typical per household rate of $10,000–$30,000 for a soil lead remediation (which involves removal of contaminated soil and replacement with clean soil; e.g., Abreu Environmental, 2024), the projected price tag for mitigating all households in the country estimated to have soil above the new USEPA standard is a staggering $290 billion to $1.2 trillion. Additionally, removing and bringing in soil mined from other places for so many millions of households seems infeasible, economics aside. Lastly, soil remediation is extremely disruptive, and if done poorly, can scatter lead contaminated soils and dust to adjacent properties and homes.

We do not think this type of mitigation is feasible at the massive scale required and we have instead focused on a more immediate, far cheaper strategy: capping current soils with clean soils and/or mulch, with potential addition of a geotextile barrier between the native soil and the capping material. At a fraction of the cost and labor of disruptive conventional soil mitigation, this approach has long been advocated by one of us (Mielke) and, although imperfect, it yields immediate and potentially life‐changing benefits for those living in these environments (e.g., Khan et al., 2021).

Covering contaminated soils rather than removing them is not a permanent solution, since the clean soil or mulch can be disturbed, exposing the lead‐contaminated soil underneath. However, even the act of covering polluted soil with clean soils will permanently dilute the lead concentration of the total soil profile if soil perturbation occurs. Given that nearly all the anthropogenic lead is captured in the upper 10 inches of soils (e.g., Filippelli & Laidlaw, 2010) adding another 10 inches of clean soil (i.e., geogenic or naturally occurring lead levels of 18–22 ppm) on top will cut the total soil lead concentration by half. Here, the adage rings true: the solution to pollution is dilution. This approach is not without its costs as well, as clean soil and/or mulch comes at a cost of about $10–$50 per cubic yard, and adding this to the dripline of a house (one yard perimeter), which consistently has the highest soil lead values (Filippelli et al., 2018) would require at least 10 cubic yards of soil. Nevertheless, this simple, relatively affordable, scalable approach provides immediate results, making it a powerful solution in this new phase of our ongoing mission to “get the lead out.”

While the general idea of capping lead polluted soils with uncontaminated soil is viable, there are locations where readily available uncontaminated soil is more difficult to come by than other areas. Thus, while effective and cheaper than entire lead remediation efforts of both soil removal and capping, a concerted effort does have to be made on a case‐by‐case basis as to the most effective means for bringing in uncontaminated capping material. In some areas, such as the Mississippi River Delta, where fresh, uncontaminated alluvium is readily available, that may be a cheap and accessible capping material. Other locations, such as New York City (NYC Office of Environmental Remediation, 2024), have invested in urban soil banks which also have the potential to mitigate soil lead exposure (Egendorf et al., 2018). However, in other areas, such as arid mining towns in the western US, other capping material like biochar, mulch, or crushed limestone may be cheaper and more readily available. Regardless of where the capping material comes from, it should undergo quick, yet effective screening to ensure it is not contaminated with lead or other heavy metals, which a handheld X‐Ray fluorescence device can easily provide within minutes.

The new USEPA guidance is specifically intended for lead in residential soils at CERCLA (i.e., Superfund) sites and RCRA (Resource Conservation and Recovery Act Corrective Action) facilities. This update will help USEPA “site teams make site‐specific cleanup decision to protect nearby communities” (USEPA, 2024b). We suggest that the USEPA expand the use of these newly proposed screening levels beyond the application of CERCLA and RCRA. Less expensive options ‐ namely capping soils with wood chips, soil, or crushed limestone—are effective alternatives to removing and replacing soil. The availability of these options might make it more economically feasible for the USEPA to make a policy of cleaning up residential soils beyond CERCLA and RCRA sites, or at least to provide guidance and subsidies to enable households to do so themselves.

## 3 Conclusions

Given the scale of the urban soil lead contamination issues and the disproportionate exposure potential faced by environmental justice communities, this issue finally needs to be fully grappled with. The USEPA has taken a critical first step by developing and implementing new soil lead screening standards—it is now up to the network of people concerned about soil lead exposure to consider reasonable, feasible, and equitable ways to reduce exposure and to regain the vitality, health, and fertility of this critical resource of the commons.

## Conflict of Interest

The authors declare no conflicts of interest relevant to this study.

## Data Availability

Raw data used to calculate soil lead percentages is available, aggregated by cities, at https://datacore.iu.edu/concern/data_sets/7d278t80h?locale=en. All other data is presented in Table 1.
